# Genetic variants of *PTPN2* are associated with lung cancer risk: a re-analysis of eight GWASs in the TRICL-ILCCO consortium

**DOI:** 10.1038/s41598-017-00850-0

**Published:** 2017-04-11

**Authors:** Yun Feng, Yanru Wang, Hongliang Liu, Zhensheng Liu, Coleman Mills, Younghun Han, Rayjean J. Hung, Yonathan Brhane, John McLaughlin, Paul Brennan, Heike Bickeboeller, Albert Rosenberger, Richard S. Houlston, Neil E. Caporaso, Maria Teresa Landi, Irene Brueske, Angela Risch, Yuanqing Ye, Xifeng Wu, David C. Christiani, Christopher I. Amos, Qingyi Wei

**Affiliations:** 1 Department of Respiration, Ruijin Hospital, School of Medicine, Shanghai Jiao Tong University, Shanghai, China; 2 Duke Cancer Institute, Duke University Medical Center, Durham, NC 27710 USA; 3 Department of Medicine, Duke University School of Medicine, Durham, NC 27710 USA; 4 Community and Family Medicine, Geisel School of Medicine, Dartmouth College, Hanover, NH 03755 USA; 5 Lunenfeld-Tanenbaum Research Institute of Mount Sinai Hospital, Toronto, Ontario Canada; 6 Public Health Ontario, Toronto, Ontario M5T 3L9 Canada; 7 Genetic Epidemiology Group, International Agency for Research on Cancer (IARC), 69372 Lyon, France; 8 Department of Genetic Epidemiology, University Medical Center, Georg-August-University Göttingen, 37073 Göttingen, Germany; 9 Division of Genetics and Epidemiology, the Institute of Cancer Research, London, SW7 3RP UK; 10 Division of Cancer Epidemiology and Genetics, National Cancer Institute, National Institutes of Health, Bethesda, MD 20892 USA; 11 Helmholtz Centre Munich, German Research Centre for Environmental Health, Institute of Epidemiology I, 85764 Neuherberg, Germany; 12 Department of Molecular Biology, University of Salzburg, 5020 Salzburg, Austria; 13 Department of Epidemiology, The University of Texas MD Anderson Cancer Center, Houston, TX 77030 USA; 14 Massachusetts General Hospital, Boston, MA 02114, USA, Department of Environmental Health, Harvard School of Public Health, Boston, MA 02115 USA

## Abstract

The T-cell protein tyrosine phosphatase (TCPTP) pathway consists of signaling events mediated by TCPTP. Mutations and genetic variants of some genes in the TCPTP pathway are associated with lung cancer risk and survival. In the present study, we first investigated associations of 5,162 single nucleotide polymorphisms (SNPs) in 43 genes of this TCPTP pathway with lung cancer risk by using summary data of six published genome-wide association studies (GWAS) of 12,160 cases and 16,838 controls. We identified 11 independent SNPs in eight genes after correction for multiple comparisons by a false discovery rate <0.20. Then, we performed *in silico* functional analyses for these 11 SNPs by eQTL analysis, two of which, *PTPN2* SNPs rs2847297 and rs2847282, were chosen as tagSNPs. We further included two additional GWAS datasets of Harvard University (984 cases and 970 controls) and deCODE (1,319 cases and 26,380 controls), and the overall effects of these two SNPs among all eight GWAS studies remained significant (OR = 0.95, 95% CI = 0.92–0.98, and *P* = 0.004 for rs2847297; OR = 0.95, 95% CI = 0.92–0.99, and *P* = 0.009 for rs2847282). In conclusion, the *PTPN2* rs2847297 and rs2847282 may be potential susceptible loci for lung cancer risk.

## Introduction

Lung cancer is one of the most common human malignancies and the leading cause of cancer-related deaths in both men and women^1^. It is estimated that 224,390 new lung cancer cases will be diagnosed in the United States in 2016^2^. Lung cancer risk likely results from joint effects and interactions of environmental and genetic factors.

Single nucleotide polymorphisms (SNPs) are the most common genetic variants and have been shown to be associated with lung cancer risk^3^. Genome-wide association studies (GWAS) have identified 30 loci in 13 genomic regions to be associated with lung cancer risk^4–15^. However, most of the SNPs identified to date have not been shown to be functional. Other approaches to GWAS including pathway-based analysis with reduced dimension or multiple testing have been emerged to identify possible functional SNPs associated with lung cancer risk.

The T-cell protein tyrosine phosphatase (TCPTP/PTPN2) is an important member of the protein-tyrosine phosphatase (PTP) family. Activating and deactivating mutations in PTP genes often result in enzymes that can either promote or suppress oncogenesis. The TCPTP pathway consists of signaling events mediated by TCPTP through negative regulation of several receptor tyrosine kinases such as epidermal growth factor receptor (EGFR)^16^, vascular endothelial growth factor receptor-2 (VEGFR2)^17^, platelet-derived growth factor receptor beta (PDGFRβ)^18^, signal transducer and activator of transcription subtypes 1 (STAT1)^19^, 3 (STAT3)^20^, and 6 (STAT6)^21^, and the insulin receptor^22^.

Studies have shown that mutations and genetic variants of some genes in the TCPTP pathway are associated with lung cancer risk and survival^23, 24^. However, SNPs in many candidate genes in the pathway have not been studied and reported. In the present study, we systematically investigated all potentially functional SNPs in TCPTP pathway genes by assessing their associations of lung cancer risk using eight published lung cancer GWAS datasets.

## Results

### Analysis of six GWAS datasets

**Figure 1:**
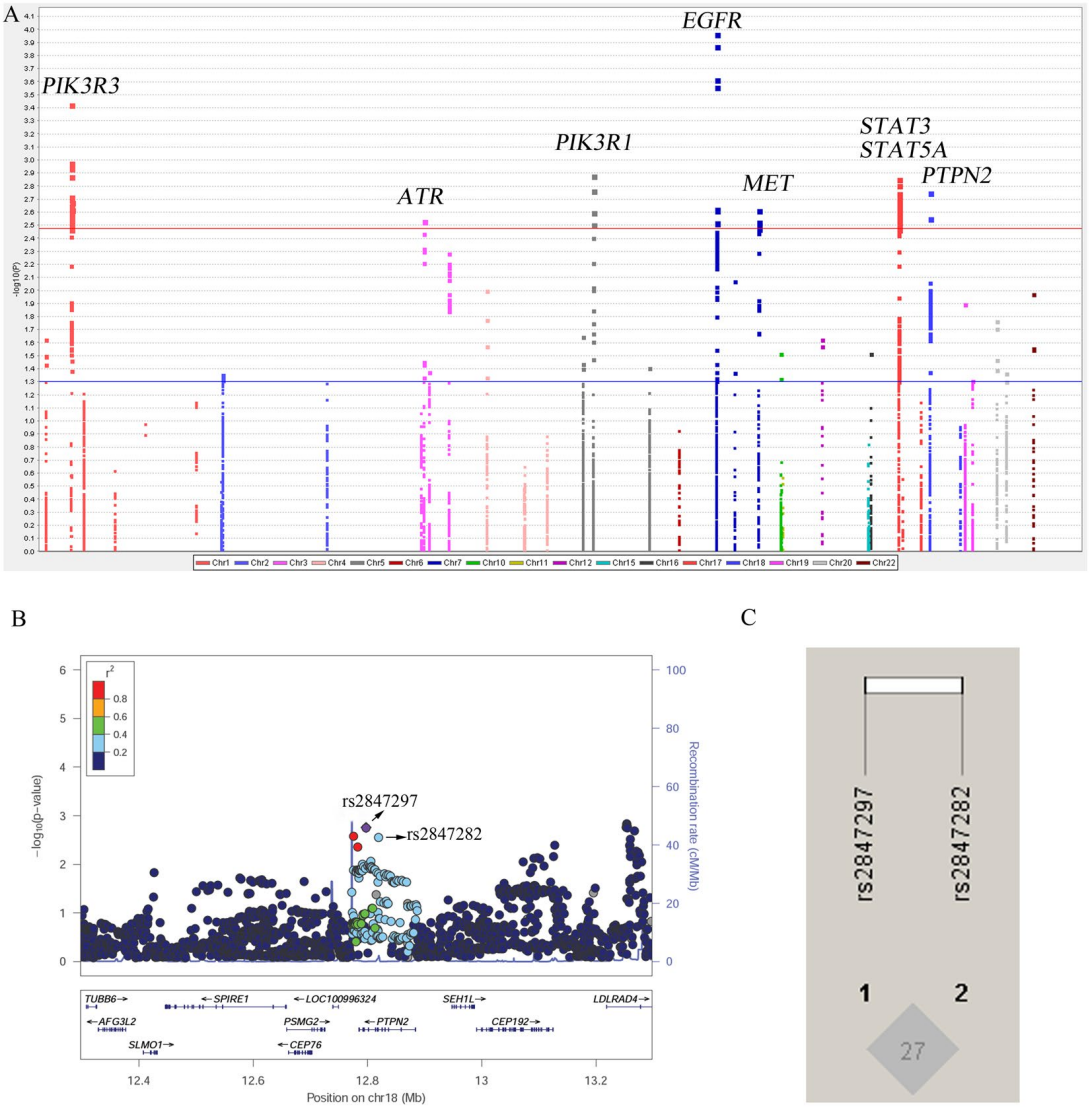
Screening of SNPs in the TCPTP pathway. (**A**) Manhattan plot of genome-wide association results of 5,162 SNPs in 43 TCPTP pathway genes and lung cancer risk in the TRICL-ILCCO Consortium. SNPs are plotted on the X-axis according to their positions on each chromosome. The association *P* values with lung cancer risk are shown on the Y-axis (as −log_10_ (*P*) values). The horizontal red line represents FDR threshold 0.20. The horizontal blue line represents *P* value of 0.05; (**B**) SNPs in *PTPN2* with 500 kb up- and downstream of the gene region and (**C**) LD plots of the SNPs in *PTPN2* with FDR <0.20. In **B**, the left-hand y-axis shows the association *P* value of each SNP, which is plotted as −log_10_ (*P*) against chromosomal base pair position; the right-hand y-axis shows the recombination rate estimated from the hg19/1000 Genomes European population.

Overall, 5162 SNPs from 43 TCPTP pathway genes in the six GWAS datasets from the Transdisciplinary Research in Cancer of the Lung and The International Lung Cancer Consortium (TRICL-ILCCO) Consortium were identified, and their associations with lung cancer risk are shown in the Manhattan plot (Fig. 1A). After multiple-testing correction, 112 SNPs in eight genes (*ATR, EGFR, MET, PIK3R1, PIK3R3, PTPN2, STAT3*, and *STAT5A*) remained significantly associated with lung cancer risk with FDR <0.20. The results of associations with lung cancer risk are summarized in Supplementary Table S2. Based on LD analysis (r^2^ > 0.30) and online functional prediction analyses by using SNPinfo, RegulomeDB, and HaploReg, we selected to perform additional analyses for 11 SNPs: rs11707731 in *ATR;* rs845553, rs1140762 and rs17172432 in *EGFR*; rs34280975 in *MET*; rs706714 in *PIK3R1;* rs7538978 in *PIK3R3;* rs2847297 and rs2847282 in *PTPN2;* rs3744483 in *STAT3;* rs1135669 in *STAT5A* for further study (Supplementary Figure S1 and Supplementary Table S3).

### Functional validation by eQTL analysis 21

**Figure 2:**
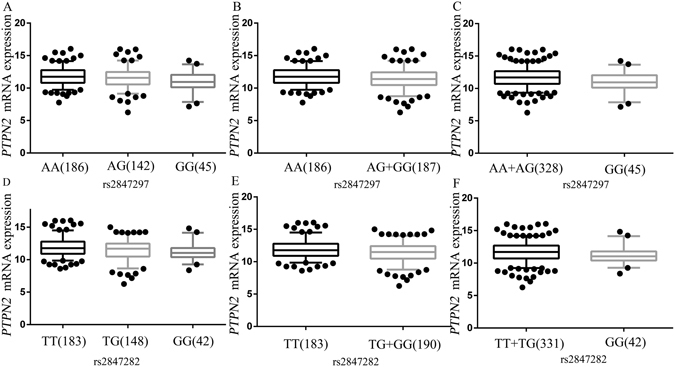
The correlations between identified SNPs and *PTPN2* mRNA expression. rs2847297 in *PTPN2* (**A**) additive model, *P* = 0.002; (**B**) dominant model, *P* = 0.017; (**C**) recessive model, *P* = 0.005) and rs2847282 in *PTPN2* (**D**), additive model, *P* = 0.0006; (**E**) dominant model, *P* = 0.001; (**F**) recessive model, *P* = 0.029).

We assessed associations between the 11 SNPs and mRNA expression levels by using the genotyping and expression data available from the lymphoblastoid cell lines derived from 373 individuals of European descent (http://www.1000genomes.org/), and we found that only rs2847297 and rs2847282 were associated with expression levels of *PTPN2* in additive, dominant and recessive models (Table 1). Regional association plots for rs2847297 and rs2847282 in 500 kb up- and downstream region were shown in Fig. 1B. The SNP rs2847297 was in a low LD with rs2847282 (Fig. 1C). *PTPN2* mRNA expression levels were significantly decreased with an increased number of the rs2847297 G allele in additive (*P* = 0.002) (Fig. 2A), dominant (*P* = 0.017) (Fig. 2B) and recessive model (*P* = 0.005) (Fig. 2C **)**. The eQTL analysis results of rs2847282 were also significant (Fig. 2D,E,F). In addition, we compared mRNA expression levels of *PTPN2* in 109 paired target tissue samples from The Cancer Genome Atlas (TCGA) and found that *PTPN2* mRNA expression levels were significantly increased in tumor tissues than normal tissues (*P* = 3.01E-05) (Supplementary Figure S2). The two SNPs rs2847297 and rs2847282 were chosen as tagSNPs, because they were significantly associated with lung cancer risk as assessed in the overall association analysis and had potential functions according to the eQTL analysis.

**Table 1:** Summary of the functional prediction and eQTL analysis results of the 11 selected SNPs in the TCPTP pathways *in silico*.

SNP	Gene	Chr.	Allele^a^	SNPinfo	Regulome DB Score	HaploReg	*P* ^b^		
							*P* (additive model)	*P* (dominant model)	*P* (recessive model)
rs7538978	*PIK3R3*	1	A/G	—	1 f	Enhancer histone marks: 9 tissues; DNAse: CRVX; Motifs changed: 6 altered motifs	0.722	0.249	0.175
rs11707731	*ATR*	3	G/T	—	4	Promoter histone marks: 4 tissues; Enhancer histone marks: 4 tissues; DNAse: ESC; Motifs changed: 4 altered motifs	0.579	0.719	0.420
rs706714	*PIK3R1*	5	A/C	TFBS	5	Enhancer histone marks: 7 tissues; DNAse: GI; Motifs changed: GATA,Nkx2,Nkx3	0.281	0.137	0.714
rs2740762	*EGFR*	7	C/A	TFBS	5	Enhancer histone marks: 13 tissues; DNAse: IPSC,MUS,PANC; Motifs changed: Foxo,NF-AT,Pax-4	0.053	0.008	0.338
rs845553	*EGFR*	7	G/A	—	4	Promoter histone marks: 4 tissues; Enhancer histone marks: 17 tissues; DNAse: 17 tissues; Proteins bound: 7; Motifs changed: 5 altered motifs	0.396	0.683	0.280
rs17172432	*EGFR*	7	T/C	—	4	Promoter histone marks: 7 tissues; Enhancer histone marks: 18 tissues; DNAse: 6 tissues; Motifs changed: 4 altered motifs	0.359	0.614	0.280
rs34280975	*MET*	7	A/G	—	2c	Enhancer histone marks: SKIN; DNAse: 4 tissues; Proteins bound: CEBPB; Motifs changed: 7 altered motifs	0.421	0.197	0.538
rs3744483	*STAT3*	17	T/C	miRNA	4	bound: 7; DNAse: 11 tissues; Motifs changed: Foxa,p300	0.907	0.856	0.467
rs1135669	*STAT5A*	17	C/T	Splicing	4	Enhancer histone marks: BLD, THYM; DNAse: OVRY,BRST; Motifs changed: BATF, Pbx3, STAT	0.062	0.079	0.255
rs2847297	*PTPN2*	18	A/G	—	—	DNAse: BLD; Motifs changed: Nkx2,Pax-5	0.005	0.017	0.005
rs2847282	*PTPN2*	18	T/G	—	5	Promoter histone marks: STRM, LIV, BLD; Enhancer histone marks: 9 tissues; DNAse: 4 tissues; Motifs changed: 26 altered motifs	0.029	0.001	0.029

^a^Reference allele/effect allele. ^b^ *P* value of eQTL analysis results

TFBS = transcription factor binding site.

### Expanded analysis by including additional two GWAS studies

**Figure 3:**
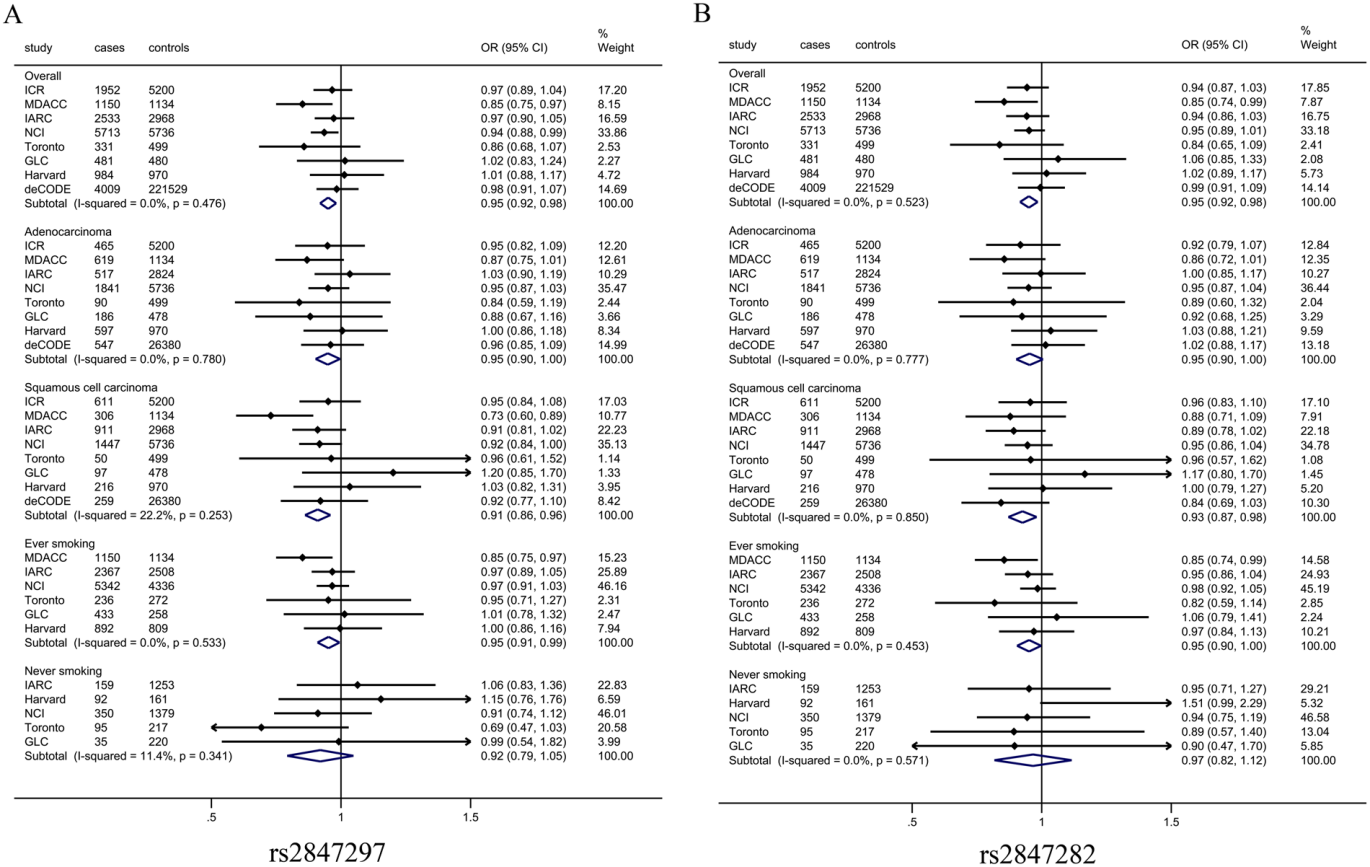
Forest plots of effect size and direction for tagSNPs from TRICL-ILCCO consortium. *PTPN2* rs2847297 *P*_combined_ = 0.004 in all individuals; *P*_combined_ = 0.052 in overall adenocarcinoma individuals; *P*_combined_ = 0.002 in overall squamous cell carcinoma individuals; *P*_combined_ = 0.042 in overall ever smoking individuals; *P*_combined_ = 0.465 in overall never smoking individuals (**A**); *PTPN2* rs2847282*P*_combined_ = 0.009 in all individuals; *P*_combined_ = 0.114 in overall adenocarcinoma individuals; *P*_combined_ = 0.016 in overall squamous cell carcinoma individuals; *P*_combined_ = 0.066 in overall ever smoking individuals; *P*_combined_ = 0.960 in overall never smoking individuals (**B**); Each box and horizontal line represent the OR point estimate and 95% CI derived from the additive model. The area of each box is proportional to the statistical weight of the study. Diamonds represent the ORs obtained from the combined analysis with 95% confidence intervals indicated by their widths. The meta-analysis includes eight GWAS studies [the Institute of Cancer Research (ICR) GWAS, the MD Anderson Cancer Center (MDACC) GWAS, the International Agency for Research on Cancer (IARC) GWAS, the National Cancer Institute (NCI) GWAS, the Lunenfeld-Tanenbaum Research Institute (Toronto) GWAS, German Lung Cancer Study (GLC) GWAS, Harvard Lung Cancer Study (Harvard) GWAS, Icelandic Lung Cancer Study (deCODE) GWAS]. NCI GWAS includes four sub-studies: the Alpha-Tocopherol, Beta-Carotene Cancer Prevention Study (ATBC), the Cancer Prevention Study II Nutrition Cohort (CPS-II), the Environment and Genetics in Lung Cancer Etiology (EAGLE), and the Prostate, Lung, Colon, Ovary Screening Trial (PLCO).

We expanded our analysis by including two additional independent lung cancer GWAS studies, Harvard Lung Cancer Study and Icelandic Lung Cancer Study (deCODE). We performed an overall meta-analysis to evaluate associations between the two *PTPN2* SNPs and lung cancer risk. We found that the overall effects among all eight GWAS studies remained significant (OR = 0.95, 95% CI = 0.92–0.98, *P*het = 0.476, and *P* = 0.004 for rs2847297; OR = 0.95, 95% CI = 0.92–0.99, *P*het = 0.523, and *P* = 0.009 for rs2847282) (Table 2 and Fig. 3A,B).

**Table 2:** Summary of the association results of two SNPs in the eight lung cancer GWAS studies.

Study population	Sample size		Imp. Quality	*PTPN2* rs2847297 A > G		Imp. Quality	*PTPN2* rs2847282 T > G	
	Cases	Controls		OR (95% CI)	*P*		OR (95% CI)	*P*
**ICR** ^1^	1952	5200	1.00	0.97 (0.89–1.04)	0.379	0.88	0.94 (0.87–1.03)	0.180
AD	465	5200	1.00	0.95(0.82–1.09)	0.459	0.87	0.92(0.79–1.07)	0.281
SQ	611	5200	1.00	0.95 (0.84–1.08)	0.425	0.87	0.96 (0.83–1.10)	0.521
**MDACC** ^2^	1150	1134	1.00	0.85 (0.75–0.97)	0.014	0.81	0.85 (0.74–0.99)	0.030
AD	619	1134	1.00	0.87 (0.75–1.01)	0.070	0.81	0.86 (0.72–1.01)	0.073
SQ	306	1134	1.00	0.73 (0.60–0.89)	0.002	0.81	0.88 (0.71–1.09)	0.246
Ever smoking	1150	1134	1.00	0.85 (0.75–0.97)	0.014	0.81	0.85 (0.74–0.99)	0.030
**IARC** ^3^	2533	3791	1.00	0.97 (0.90–1.05)	0.475	0.77	0.94 (0.86–1.03)	0.188
AD	517	2824	1.00	1.03 (0.90–1.19)	0.641	0.77	1.00 (0.85–1.17)	0.961
SQ	911	2968	1.00	0.91 (0.81–1.02)	0.104	0.77	0.89 (0.78–1.02)	0.084
Ever smoking	2367	2508	1.00	0.97 (0.89–1.05)	0.446	0.77	0.95 (0.86–1.04)	0.273
Never smoking	159	1253	1.00	1.06 (0.83–1.36)	0.623	0.77	0.95 (0.71–1.27)	0.735
**NCI** ^4^	5713	5736	1.00	0.94 (0.88–0.99)	0.022	0.87	0.95 (0.89–1.01)	0.116
AD	1841	5736	1.00	0.95 (0.87–1.03)	0.225	0.87	0.95 (0.87–1.04)	0.257
SQ	1447	5736	1.00	0.92 (0.84–1.00)	0.060	0.88	0.95 (0.86–1.04)	0.258
Ever smoking	5342	4336	1.00	0.97(0.91–1.03)	0.297	0.88	0.98 (0.92–1.06)	0.649
Never smoking	350	1379	1.00	0.91(0.74–1.12)	0.376	0.88	0.94 (0.75–1.19)	0.622
**Toronto** ^5^	331	499	1.00	0.86 (0.68–1.07)	0.182	0.85	0.84 (0.65–1.09)	0.180
AD	90	499	1.00	0.84 (0.59–1.19)	0.326	0.85	0.89 (0.60–1.32)	0.566
SQ	50	499	1.00	0.96 (0.61–1.52)	0.870	0.85	0.96 (0.57–1.62)	0.871
Ever smoking	236	272	1.00	0.95(0.71–1.27)	0.735	0.87	0.82 (0.59–1.14)	0.231
Never smoking	95	217	1.00	0.69(0.47–1.03)	0.065	0.87	0.89 (0.57–1.40)	0.611
**GLC** ^6^	481	478	1.00	1.01 (0.83–1.24)	0.881	0.80	1.06 (0.85–1.33)	0.584
AD	186	478	1.00	0.88 (0.67–1.16)	0.368	0.80	0.92 (0.68–1.25)	0.609
SQ	97	478	1.00	1.20 (0.85–1.70)	0.299	0.80	1.17 (0.80–1.70)	0.426
Ever smoking	433	258	1.00	1.01 (0.78–1.32)	0.920	0.80	1.06 (0.79–1.41)	0.701
Never smoking	35	220	1.00	0.99 (0.54–1.82)	0.978	0.80	0.90 (0.47–1.70)	0.736
**Discovery combined**	12160	16838		0.94 (0.91–0.98)	0.002		0.94 (0.90–0.98)	0.003
**Harvard** ^7^	984	970	1.00	1.01 (0.88–1.17)	0.857	0.83	1.02 (0.89–1.17)	0.791
AD	597	970	1.00	1.00 (0.86–1.18)	0.952	0.83	1.04 (0.88–1.21)	0.673
SQ	216	970	1.00	1.03 (0.82–1.31)	0.781	0.83	1.00 (0.79–1.27)	0.967
Ever smoking	892	809	1.00	1.00 (0.86–1.16)	0.962	0.83	0.97 (0.84–1.13)	0.687
Never smoking	92	161	1.00	1.15 (0.76–1.76)	0.502	0.83	1.51 (0.99–2.29)	0.053
**deCOD** ^8^	1319	26380	1.00	0.98 (0.91–1.07)	0.689	0.89	0.99 (0.91–1.09)	0.911
AD	547	26380	1.00	0.96 (0.85–1.09)	0.524	0.89	1.02 (0.88–1.17)	0.834
SQ	259	26380	1.00	0.92 (0.77–1.10)	0.373	0.89	0.84 (0.69–1.03)	0.095
**Replication combined**	2303	27350		0.99 (0.92–1.06)	0.803		1.00 (0.93–1.08)	0.960
**Overall**	14463	44188		0.95 (0.92–0.98)	0.004		0.95 (0.92–0.99)	0.009
**Overall AD combined**	4862	43221		0.95 (0.91–1.00)	0.053		0.96 (0.91–1.01)	0.114
**Overall SQ combined**	3897	43365		0.92 (0.87–0.97)	0.002		0.93 (0.88–0.99)	0.016
**Overall ever smoking combined**	10420	9317		0.96 (0.91–1.00)	0.043		0.96 (0.91–1.00)	0.064
**Overall never smoking combined**	731	3230		0.95 (0.83–1.09)	0.467		1.00 (0.86–1.16)	0.959

AD: adenocarcinoma, SQ: squamous cell carcinoma. The combined OR and *P* value were estimated using a fixed-effects model.

^1^ICR: the Institute of Cancer Research Genome-wide Association Study, UK.

^2^MDACC: the MD Anderson Cancer Center Genome-wide Association Study, US.

^3^IARC: the International Agency for Research on Cancer Genome-wide Association Study, France.

^4^NCI: the National Cancer Institute Genome-wide Association Study, US.

^5^Toronto: the Samuel Lunenfeld Research Institute Genome-wide Association Study, Toronto, Canada.

^6^GLC: German Lung Cancer Study, Germany.

^7^Harvard: Harvard Lung Cancer Study, USA.

^8^deCODE: Icelandic Lung Cancer Study, Iceland.

In subgroup analysis by histology (Table 2, Fig. 3), we found that the rs2847297 G allele was borderline associated with lung adenocarcinoma (AD) risk (OR = 0.95, 95% CI = 0.91–1.00, *P* = 0.052) and significantly associated with squamous cell lung carcinoma (SQ) risk (OR = 0.92, 95% CI = 0.87–0.97, *P* = 0.002, Fig. 3A). We also found the rs2847282 G allele was associated with SQ risk (OR = 0.93, 95% CI = 0.88–0.99, *P* = 0.016), while there was no statistical association with AD risk (OR = 0.96, 95% CI = 0.91–1.01, *P* = 0.114, Fig. 3B). In subgroup analysis by smoking status, there was a marginal significant decrease in lung cancer risk for the rs2847297 G allele among ever smokers (OR = 0.96, 95% CI = 0.91–1.00, *P* = 0.042), but not among never smokers (OR = 0.95, 95% CI = 0.83–1.09, *P* = 0.465, Fig. 3A). However, there was no association with the *PTPN2* rs2847282 G allele and lung cancer risk among ever smokers (OR = 0.96, 95% CI = 0.91–1.00, *P* = 0.066 and never smokers (OR = 1.00, 95% CI = 0.86–1.16, *P* = 0.960, Fig. 3B).

## Discussion

In the present study, we sought to investigate associations between genetic variants in the TCPTP pathway genes and lung cancer risk using eight published GWAS studies of 14,463 cases and 44,188 controls. The principal findings included two novel, potentially functional SNPs, rs2847297 and rs2847282 of *PTPN2*, that were both associated with a decreased lung cancer risk and a decreased mRNA expression level of *PTPN2*, particularly in subgroups of ever smokers and squamous cell lung carcinoma. Four articles about pathway-based analysis and lung cancer risk (Centrosome, DNA repair, lncRNA and RNA degradation) have been accepted or published in our laboratory. We found that the loci of two SNPs in *PTPN2* were different from previous studies in our lab and GWAS studies.

PTPN2 plays a dual role in development and progression of cancer. Proliferation and cell cycle assays demonstrated that overexpression of PTPN2 would decrease serum requirement, increase formation of larger colonies in soft agar, alter morphology, and rapidly progress through G1 and S phases and the rate of cell division^25, 26^. Another study showed that the proliferation rate would reduce in TCPTP (−/−), compared to TCPTP (+/+), lymphocytes^27^. We found that *PTPN2* mRNA expression levels in matched lung cancer tissues were increased compared to adjacent normal tissues from the TCGA database, some other studies also demonstrated that PTPN2 expression levels were higher in lung AD^28, 29^ and SQ^30, 31^ than in normal lung tissues. These findings provided oncogenic evidence of *PTPN2* and were consistent with our results that the two susceptibility loci of *PTPN2* were associated with a decreased lung cancer risk as a result of a decreased mRNA expression level of the gene. In addition, we found that the eQTL analysis result of rs2847297 in lung tissue was also significant in the GTEx analysis (*P* = 4.0E10–7) (http://www.gtexportal.org/home/eqtls/bySnp?snpId=rs2847297&tissueName=All). This result is also consistent with the eQTL analysis from the lymphoblastoid cell lines in the present study. However, it has been reported that overexpression of PTPN2 induces apoptosis in the p53 + A549 and MCF-7 cells but not in p53- HeLa cells, also consistent with features of a tumor suppressor^32^. Another study demonstrated that PTPN2 was absent in a large proportion of “triple-negative” primary human breast cancers and PTPN2 overexpression would suppress tumor growth^33^.

In subgroup analysis we found that the two SNPs were more likely to be associated with SQ risk, and the risk associated with rs2847297 G allele was more likely to be among ever smoking. Cigarette smoke is the major risk factor for lung cancer, especially for SQ. Study showed that smoking led to an increased expression of Nkx2^34^, which is the transcription factor (TF) of *PTPN2*. Therefore, it is likely that the locus has the possibility of influencing lung cancer risk of ever smokers through changing the expression of PTPN2.

Our study has some limitations. First, genes in the TCPTP pathway were identified mainly from the Molecular Signatures Database and Genecards. Although we did search some relative articles to complete the list of genes in the pathway, some newly discovered genes in the pathway might have been missed. Second, although we demonstrated the association of thetwo novel potentially functional loci in *PTPN2* with lung cancer risk with functional evidence from eQTL analyses, the exact biochemical and molecular mechanisms are still unclear. Third, our eQTL analyses were limited to publicly available data from lymphoblastoid cell lines but target tissues, which could provide more direct correlation results between the two SNPs and PTPN2 expression.

Taken together, the present study revealed two novel, potentially functional susceptibility loci in *PTPN2* associated with lung cancer risk in European populations, particularly among ever smokers and squamous carcinoma. Further validation and functional evaluation of these genetic variants are warranted to verify our findings.

## Materials and Methods

### Study populations

The present study first used genotyping data from the TRICL-ILCCO consortium, which included 12,160 lung cancer cases and 16,838 controls (all Europeans) of six previously published GWAS studies: The University of Texas MD Anderson Cancer Center (MDACC), Institute of Cancer Research (ICR), National Cancer Institute (NCI), International Agency for Research on Cancer (IARC), Toronto study from Samuel Lunenfeld Research Institute study (Toronto), and German Lung Cancer Study (GLC). The expanded analysis included additional two GWAS studies of European ancestry from the Harvard Lung Cancer Study (984 cases and 970 controls)^35^ and the Icelandic Lung Cancer Study (deCODE) (1,319 cases and 26,380 controls)^36^ of the ILCCO. Details of the study populations are presented in the supplementary file. A written informed consent was obtained by all participating GWAS studies. All methods were performed in accordance with the relevant guidelines and regulations for each of the participating institutions, and the present study followed the study protocols approved by Duke University Health System Institutional Review Board.

### Selection of Genes and SNPs from TCPTP pathway

**Figure 4:**
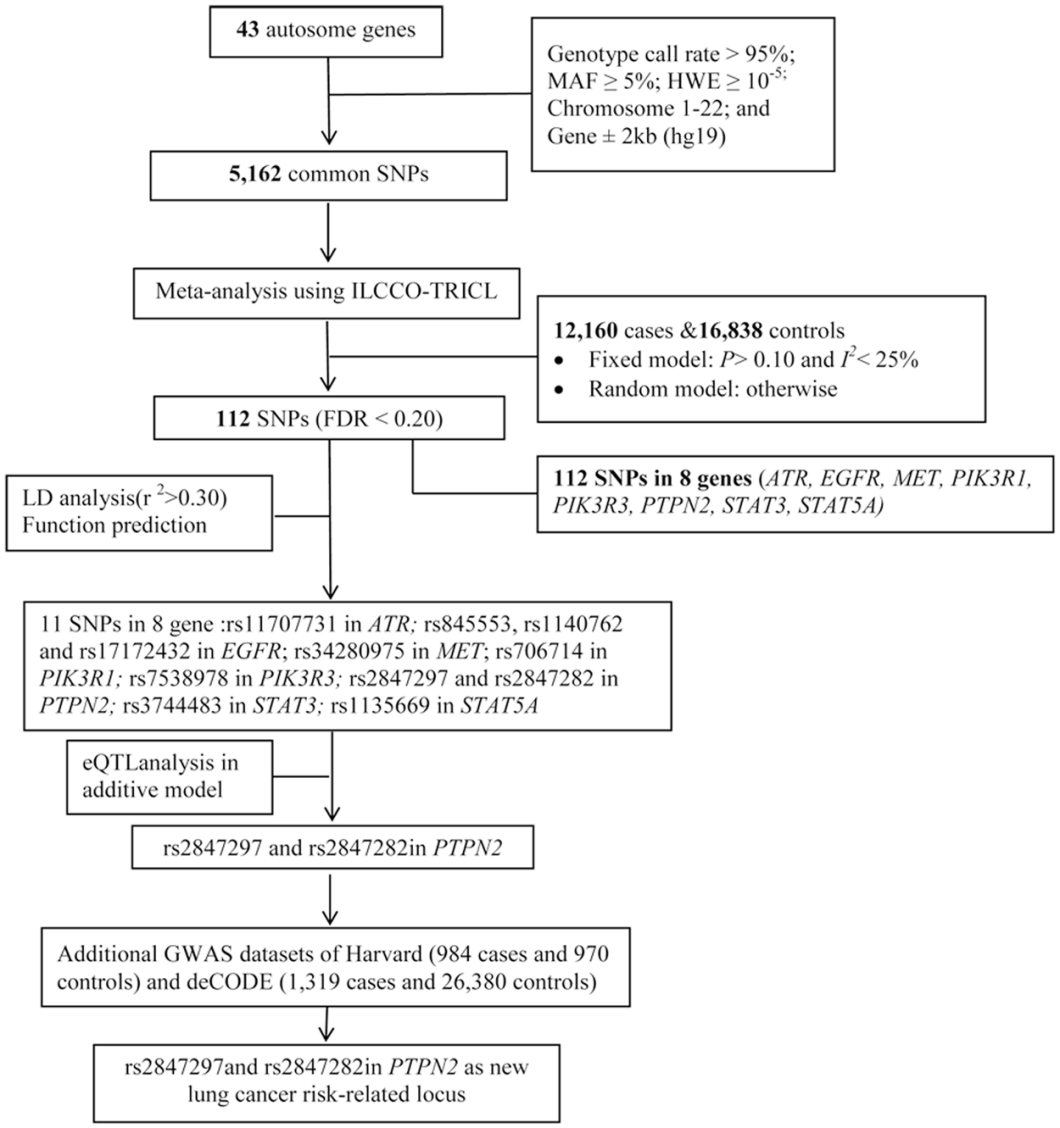
Flowchart of SNP selection among the TCPTP pathway genes.

Genotyping in these GWAS studies was performed by one of Illumina HumanHap 317, 317 + 240 S, 370Duo, 550, 610 or 1 M arrays. IMPUTE2 v2.1.1 or MaCH v1.0 software was used for imputation. Genes in the TCPTP pathway were identified from the Molecular Signatures Database (http://www.broadinstitute.org/gsea/index.jsp)^37^ and Genecards (http://www.genecards.org/). Overall, 43 genes located on autosomal chromosomes were selected (detailed in Supplementary Table S1). The final meta-analysis contained 5,162 SNPs with the following inclusion criteria: genotyping rate >95%, minor allele frequency (MAF) ≥ 5%, and Hardy-Weinberg Equilibrium (HWE) exact *P* value ≥ 10^−5^. The detailed workflow is shown in Fig. 4.

### *In silico* functional prediction and validation

We use three *in silico* tools, SNPinfo (http://snpinfo.niehs.nih.gov/snpinfo/snpfunc.htm)^38^, RegulomeDB (http://regulomedb.org/)^39^, and HaploReg (http://www.broadinstitute.org/mammals/haploreg/haploreg.php)^40^ to predict potential functions. The expression quantitative trait loci (eQTL) analysis was performed in the 1000 Genomes Project^41^. The mRNA expression of lung cancer tissue samples was performed in TCGA^42^.

### Statistical analysis

Odds ratios (ORs) and their 95% confidence intervals (CIs) were calculated using Stata (v10, State College, Texas, USA) and PLINK (v1.06) software. A meta-analysis with the inverse variance method was employed on the 5,162 SNPs. We used Cochran’s Q statistic to test for heterogeneity and I^2^ statistic for the proportion of the total variation^43^. The fixed-effects model was used when there was no heterogeneity among GWAS studies (Q-test P > 0.100 and I2 < 25%); otherwise, the random-effects model was used. The false discovery rate (FDR) was performed to control for multiple testing with a threshold <0.20^44^. The genes mRNA expression levels in lung cancer and adjacent tissues from TCGA database were performed by paired t-test. Regional association plots were performed by LocusZoom^45^. Haploview v4.2 was used to generate the Manhattan plot and LD plots^46^. All other analyses were conducted with SAS (Version 9.3; SAS Institute, Cary, NC, USA).

## Electronic supplementary material

Supplementary information
