# Supplementary material for: Genetic variants of PTPN2 are associated with lung cancer risk: a re-analysis of eight GWASs in the TRICL-ILCCO consortium
Source: Sci Rep. 2017 Apr 11;7:825. doi: 10.1038/s41598-017-00850-0 (PMC5429754; doi:10.1038/s41598-017-00850-0)
Supplement: Supplementary file 1 — Supplementary information [file 41598_2017_850_MOESM1_ESM.doc]

**Supplementary information to:**

**Genetic variants of *PTPN2* are associated with lung cancer risk: a re-analysis of eight GWASs in the TRICL-ILCCO consortium**

Yun Feng, Yanru Wang, Hongliang Liu, Zhensheng Liu, Coleman Mills, Younghun Han, Rayjean J. Hung, Yonathan Brhane, John McLaughlin, Paul Brennan, Heike Bickeboeller, Albert Rosenberger, Richard S. Houlston, Neil E. Caporaso, Maria Teresa Landi, Irene Brueske, Angela Risch, Yuanqing Ye, Xifeng Wu, David C. Christiani, Christopher I. Amos, Qingyi Wei

**Contents**

**Supplementary table S1. .………………………………….……………….……………………2**

**Supplementary table S2. …………………………………………………………………………3**

**Supplementary table S3. ……………………………………….…………………………………6**

**Supplementary figure S1. ……………………………………….………………………………..8**

**Supplementary figure S2. ……………………………………….………………………………..9**

**Supplementary file……………………………………….………………………………………...10**

| **Supplementary Table S1.** The selective results and criteria of the TCPTP pathways | | |
| --- | --- | --- |
| Dataset | Name of Pathway | Genes |
| GO | NO DATA | 0 |
| Reactome | NO DATA | 0 |
| KEGG | NO DATA | 0 |
| PID | PID_TCPTP_PATHWAY | 43 |
| BioCarta | NO DATA | 0 |
| GO, gene ontology; KEGG, Kyoto encyclopedia of genes and genomes; PID, pathway interaction database.  Keyword: TCPTP;  Collection: Canonical pathways + GO gene sets;  Organism: Homo sapiens;  Contributor: All contributors. | | |

| **Supplementary Table S2**. Associations between 112 SNPs in the TCPTP pathways and lung cancer risk with FDR < 0.20 | | | | | | | | | | |
| --- | --- | --- | --- | --- | --- | --- | --- | --- | --- | --- |
| SNP | Gene | Chr. | Position (hg19) | Allele^a^ | EAF | Q^b^ | *I*^2^ | OR (95%CI) | *P* | FDR |
| rs4660342 | *PIK3R3* | 1 | 46595082 | G/T | 0.38 | 0.76 | 0 | 0.93 (0.90-0.97) | 3.72E-04 | 0.168 |
| rs7538978 | *PIK3R3* | 1 | 46505054 | A/G | 0.28 | 0.75 | 0 | 0.94 (0.90-0.97) | 1.04E-03 | 0.168 |
| rs785498 | *PIK3R3* | 1 | 46592414 | T/C | 0.29 | 0.76 | 0 | 0.94 (0.90-0.97) | 1.13E-03 | 0.168 |
| rs809774 | *PIK3R3* | 1 | 46560244 | A/T | 0.29 | 0.73 | 0 | 0.94 (0.90-0.98) | 1.14E-03 | 0.168 |
| rs785508 | *PIK3R3* | 1 | 46527159 | A/G | 0.29 | 0.75 | 0 | 0.94 (0.90-0.98) | 1.15E-03 | 0.168 |
| rs785509 | *PIK3R3* | 1 | 46527300 | A/G | 0.29 | 0.75 | 0 | 0.94 (0.90-0.98) | 1.15E-03 | 0.168 |
| rs785513 | *PIK3R3* | 1 | 46538320 | C/T | 0.28 | 0.72 | 0 | 0.94 (0.90-0.98) | 1.32E-03 | 0.168 |
| rs785518 | *PIK3R3* | 1 | 46568422 | T/A | 0.32 | 0.75 | 0 | 0.94 (0.90-0.98) | 1.88E-03 | 0.168 |
| rs785501 | *PIK3R3* | 1 | 46579611 | T/C | 0.29 | 0.72 | 0 | 0.94 (0.91-0.98) | 1.98E-03 | 0.168 |
| rs1707303 | *PIK3R3* | 1 | 46598273 | C/A | 0.29 | 0.74 | 0 | 0.94 (0.90-0.98) | 2.08E-03 | 0.168 |
| rs814168 | *PIK3R3* | 1 | 46591623 | G/A | 0.29 | 0.65 | 0 | 0.94 (0.91-0.98) | 2.20E-03 | 0.168 |
| rs785500 | *PIK3R3* | 1 | 46593044 | T/C | 0.30 | 0.71 | 0 | 0.94 (0.90-0.98) | 2.30E-03 | 0.168 |
| rs785504 | *PIK3R3* | 1 | 46595696 | T/C | 0.28 | 0.77 | 0 | 0.94 (0.91-0.98) | 2.36E-03 | 0.168 |
| rs785506 | *PIK3R3* | 1 | 46540652 | T/C | 0.28 | 0.70 | 0 | 0.94 (0.91-0.98) | 2.40E-03 | 0.168 |
| rs785486 | *PIK3R3* | 1 | 46575148 | C/G | 0.28 | 0.69 | 0 | 0.94 (0.91-0.98) | 2.47E-03 | 0.168 |
| rs785512 | *PIK3R3* | 1 | 46534543 | G/A | 0.28 | 0.72 | 0 | 0.94 (0.91-0.98) | 2.51E-03 | 0.168 |
| rs1707337 | *PIK3R3* | 1 | 46508769 | G/A | 0.28 | 0.73 | 0 | 0.94 (0.91-0.98) | 2.51E-03 | 0.168 |
| rs785490 | *PIK3R3* | 1 | 46577124 | C/T | 0.28 | 0.71 | 0 | 0.94 (0.91-0.98) | 2.52E-03 | 0.168 |
| rs9429186 | *PIK3R3* | 1 | 46539687 | T/C | 0.28 | 0.75 | 0 | 0.94 (0.91-0.98) | 2.55E-03 | 0.168 |
| rs1768807 | *PIK3R3* | 1 | 46510570 | G/A | 0.28 | 0.72 | 0 | 0.94 (0.91-0.98) | 2.60E-03 | 0.168 |
| rs1613296 | *PIK3R3* | 1 | 46546852 | G/T | 0.28 | 0.73 | 0 | 0.94 (0.91-0.98) | 2.61E-03 | 0.168 |
| rs1768802 | *PIK3R3* | 1 | 46547868 | G/A | 0.28 | 0.73 | 0 | 0.94 (0.91-0.98) | 2.61E-03 | 0.168 |
| rs1588663 | *PIK3R3* | 1 | 46548825 | A/G | 0.28 | 0.72 | 0 | 0.94 (0.91-0.98) | 2.62E-03 | 0.168 |
| rs1768800 | *PIK3R3* | 1 | 46549188 | G/A | 0.28 | 0.72 | 0 | 0.94 (0.91-0.98) | 2.62E-03 | 0.168 |
| rs1768801 | *PIK3R3* | 1 | 46549137 | G/C | 0.28 | 0.72 | 0 | 0.94 (0.91-0.98) | 2.62E-03 | 0.168 |
| rs785507 | *PIK3R3* | 1 | 46541558 | T/C | 0.28 | 0.74 | 0 | 0.94 (0.91-0.98) | 2.63E-03 | 0.168 |
| rs1768818 | *PIK3R3* | 1 | 46514286 | C/G | 0.28 | 0.73 | 0 | 0.94 (0.91-0.98) | 2.64E-03 | 0.168 |
| rs1768817 | *PIK3R3* | 1 | 46514534 | G/C | 0.28 | 0.73 | 0 | 0.94 (0.91-0.98) | 2.64E-03 | 0.168 |
| rs1707339 | *PIK3R3* | 1 | 46513179 | G/A | 0.28 | 0.73 | 0 | 0.94 (0.91-0.98) | 2.66E-03 | 0.168 |
| rs1085240 | *PIK3R3* | 1 | 46523978 | A/G | 0.28 | 0.75 | 0 | 0.94 (0.91-0.98) | 2.66E-03 | 0.168 |
| rs785470 | *PIK3R3* | 1 | 46519622 | A/C | 0.28 | 0.73 | 0 | 0.94 (0.91-0.98) | 2.66E-03 | 0.168 |
| rs785465 | *PIK3R3* | 1 | 46522577 | C/A | 0.28 | 0.73 | 0 | 0.94 (0.91-0.98) | 2.67E-03 | 0.168 |
| rs785468 | *PIK3R3* | 1 | 46521517 | A/G | 0.28 | 0.73 | 0 | 0.94 (0.91-0.98) | 2.67E-03 | 0.168 |
| rs1707317 | *PIK3R3* | 1 | 46510642 | T/C | 0.28 | 0.74 | 0 | 0.94 (0.91-0.98) | 2.67E-03 | 0.168 |
| rs1768815 | *PIK3R3* | 1 | 46528603 | A/G | 0.28 | 0.74 | 0 | 0.94 (0.91-0.98) | 2.67E-03 | 0.168 |
| rs785493 | *PIK3R3* | 1 | 46584859 | G/A | 0.28 | 0.74 | 0 | 0.94 (0.91-0.98) | 2.68E-03 | 0.168 |
| rs2297883 | *PIK3R3* | 1 | 46511487 | C/G | 0.28 | 0.73 | 0 | 0.94 (0.91-0.98) | 2.69E-03 | 0.168 |
| rs1707338 | *PIK3R3* | 1 | 46511981 | G/T | 0.28 | 0.73 | 0 | 0.94 (0.91-0.98) | 2.69E-03 | 0.168 |
| rs1707322 | *PIK3R3* | 1 | 46505147 | G/A | 0.28 | 0.69 | 0 | 0.94 (0.91-0.98) | 2.69E-03 | 0.168 |
| rs1612419 | *PIK3R3* | 1 | 46546945 | C/T | 0.28 | 0.74 | 0 | 0.94 (0.91-0.98) | 2.69E-03 | 0.168 |
| rs785469 | *PIK3R3* | 1 | 46521091 | T/C | 0.28 | 0.73 | 0 | 0.94 (0.91-0.98) | 2.69E-03 | 0.168 |
| rs785497 | *PIK3R3* | 1 | 46591903 | C/G | 0.28 | 0.74 | 0 | 0.94 (0.91-0.98) | 2.70E-03 | 0.168 |
| rs785463 | *PIK3R3* | 1 | 46524234 | A/G | 0.28 | 0.75 | 0 | 0.94 (0.91-0.98) | 2.75E-03 | 0.168 |
| rs785466 | *PIK3R3* | 1 | 46521792 | A/G | 0.28 | 0.75 | 0 | 0.94 (0.91-0.98) | 2.75E-03 | 0.168 |
| rs785483 | *PIK3R3* | 1 | 46559171 | C/T | 0.28 | 0.70 | 0 | 0.94 (0.91-0.98) | 2.76E-03 | 0.168 |
| rs785510 | *PIK3R3* | 1 | 46528618 | C/T | 0.28 | 0.75 | 0 | 0.94 (0.91-0.98) | 2.76E-03 | 0.168 |
| rs785467 | *PIK3R3* | 1 | 46521559 | T/A | 0.28 | 0.75 | 0 | 0.94 (0.91-0.98) | 2.78E-03 | 0.168 |
| rs2458400 | *PIK3R3* | 1 | 46530798 | T/C | 0.28 | 0.72 | 0 | 0.94 (0.91-0.98) | 2.91E-03 | 0.168 |
| rs796773 | *PIK3R3* | 1 | 46564475 | T/C | 0.28 | 0.70 | 0 | 0.94 (0.91-0.98) | 2.94E-03 | 0.168 |
| rs785519 | *PIK3R3* | 1 | 46568562 | T/C | 0.28 | 0.70 | 0 | 0.94 (0.91-0.98) | 2.95E-03 | 0.168 |
| rs785484 | *PIK3R3* | 1 | 46574015 | C/T | 0.28 | 0.71 | 0 | 0.94 (0.91-0.98) | 3.06E-03 | 0.168 |
| rs785517 | *PIK3R3* | 1 | 46567535 | T/A | 0.28 | 0.72 | 0 | 0.94 (0.91-0.98) | 3.07E-03 | 0.168 |
| rs785516 | *PIK3R3* | 1 | 46564758 | G/T | 0.28 | 0.72 | 0 | 0.94 (0.91-0.98) | 3.09E-03 | 0.168 |
| rs785496 | *PIK3R3* | 1 | 46588006 | C/T | 0.28 | 0.68 | 0 | 0.94 (0.91-0.98) | 3.34E-03 | 0.172 |
| rs785462 | *PIK3R3* | 1 | 46525751 | A/G | 0.31 | 0.84 | 0 | 0.94 (0.91-0.98) | 3.80E-03 | 0.180 |
| rs11707731 | *ATR* | 3 | 142287627 | G/T | 0.20 | 0.66 | 0 | 0.94 (0.90-0.98) | 2.91E-03 | 0.168 |
| rs68065420 | *ATR* | 3 | 142289863 | C/A | 0.20 | 0.64 | 0 | 0.94 (0.90-0.98) | 3.65E-03 | 0.180 |
| rs13156223 | *PIK3R1* | 5 | 67525540 | G/C | 0.23 | 0.49 | 0 | 0.93 (0.90-0.97) | 1.31E-03 | 0.168 |
| rs706714 | *PIK3R1* | 5 | 67522851 | A/C | 0.25 | 0.51 | 0 | 0.94 (0.90-0.98) | 1.69E-03 | 0.168 |
| rs10515070 | *PIK3R1* | 5 | 67525575 | T/A | 0.25 | 0.55 | 0 | 0.94 (0.90-0.98) | 2.47E-03 | 0.168 |
| rs706713 | *PIK3R1* | 5 | 67522722 | C/T | 0.24 | 0.59 | 0 | 0.94 (0.90-0.98) | 3.09E-03 | 0.168 |
| rs173701 | *PIK3R1* | 5 | 67512124 | G/C | 0.22 | 0.25 | 25 | 0.94 (0.89-0.99) | 3.90E-03 | 0.183 |
| rs845559 | *EGFR* | 7 | 55247960 | C/T | 0.15 | 0.71 | 0 | 0.91 (0.87-0.95) | 1.07E-04 | 0.168 |
| rs2692456 | *EGFR* | 7 | 55272826 | A/G | 0.17 | 0.94 | 0 | 0.91 (0.87-0.96) | 1.33E-04 | 0.168 |
| rs2740762 | *EGFR* | 7 | 55261342 | C/A | 0.17 | 0.88 | 0 | 0.92 (0.88-0.96) | 2.38E-04 | 0.168 |
| rs940805 | *EGFR* | 7 | 55251807 | C/T | 0.16 | 0.71 | 0 | 0.92 (0.87-0.96) | 2.72E-04 | 0.168 |
| rs845553 | *EGFR* | 7 | 55245717 | G/A | 0.15 | 0.57 | 0 | 0.93 (0.88-0.97) | 2.35E-03 | 0.168 |
| rs845554 | *EGFR* | 7 | 55246664 | G/C | 0.15 | 0.52 | 0 | 0.93 (0.88-0.97) | 2.38E-03 | 0.168 |
| rs1140475 | *EGFR* | 7 | 55266417 | C/T | 0.12 | 0.99 | 0 | 0.92 (0.87-0.97) | 2.41E-03 | 0.168 |
| rs17172432 | *EGFR* | 7 | 55141317 | T/C | 0.18 | 0.70 | 0 | 1.07 (1.02-1.12) | 2.98E-03 | 0.168 |
| rs12532468 | *EGFR* | 7 | 55248231 | G/A | 0.40 | 0.27 | 22 | 0.95 (0.91-1.00) | 3.02E-03 | 0.168 |
| rs2075101 | *EGFR* | 7 | 55250026 | G/A | 0.41 | 0.25 | 24 | 0.95 (0.91-1.00) | 3.53E-03 | 0.177 |
| rs13243364 | *EGFR* | 7 | 55248488 | A/G | 0.41 | 0.27 | 22 | 0.95 (0.91-1.00) | 3.54E-03 | 0.177 |
| rs1050171 | *EGFR* | 7 | 55249063 | A/G | 0.41 | 0.34 | 12 | 0.95 (0.92-0.99) | 3.79E-03 | 0.180 |
| rs6944906 | *EGFR* | 7 | 55251953 | A/G | 0.41 | 0.30 | 18 | 0.95 (0.92-0.99) | 4.01E-03 | 0.186 |
| rs845560 | *EGFR* | 7 | 55250794 | C/T | 0.26 | 0.53 | 0 | 0.94 (0.91-0.98) | 4.28E-03 | 0.197 |
| rs2283053 | *MET* | 7 | 116427019 | A/G | 0.19 | 0.82 | 0 | 0.93 (0.89-0.98) | 2.41E-03 | 0.168 |
| rs2299440 | *MET* | 7 | 116416327 | C/T | 0.19 | 0.81 | 0 | 0.94 (0.90-0.98) | 2.92E-03 | 0.168 |
| rs2073560 | *MET* | 7 | 116423161 | G/A | 0.19 | 0.82 | 0 | 0.94 (0.90-0.98) | 3.14E-03 | 0.169 |
| rs13239139 | *MET* | 7 | 116435123 | A/G | 0.19 | 0.80 | 0 | 0.94 (0.90-0.98) | 3.25E-03 | 0.172 |
| rs3807997 | *MET* | 7 | 116420558 | C/A | 0.19 | 0.82 | 0 | 0.94 (0.90-0.98) | 3.29E-03 | 0.172 |
| rs35314984 | *MET* | 7 | 116431948 | T/G | 0.19 | 0.82 | 0 | 0.94 (0.90-0.98) | 3.31E-03 | 0.172 |
| rs34280975 | *MET* | 7 | 116430265 | A/G | 0.19 | 0.83 | 0 | 0.94 (0.90-0.98) | 3.60E-03 | 0.179 |
| rs9909736 | *STAT3* | 17 | 40470342 | A/C | 0.20 | 0.61 | 0 | 0.93 (0.89-0.97) | 1.54E-03 | 0.168 |
| rs3809758 | *STAT3* | 17 | 40471980 | C/T | 0.20 | 0.64 | 0 | 0.93 (0.89-0.97) | 1.82E-03 | 0.168 |
| rs9891867 | *STAT3* | 17 | 40468489 | G/A | 0.18 | 0.43 | 0 | 0.93 (0.89-0.97) | 1.87E-03 | 0.168 |
| rs8066464 | *STAT3* | 17 | 40471101 | T/A | 0.20 | 0.64 | 0 | 0.93 (0.89-0.97) | 1.88E-03 | 0.168 |
| rs8074524 | *STAT3* | 17 | 40469598 | C/T | 0.20 | 0.64 | 0 | 0.93 (0.89-0.98) | 1.90E-03 | 0.168 |
| rs3744483 | *STAT3* | 17 | 40466438 | T/C | 0.20 | 0.62 | 0 | 0.93 (0.89-0.98) | 1.94E-03 | 0.168 |
| rs9909659 | *STAT3* | 17 | 40473835 | G/A | 0.20 | 0.62 | 0 | 0.93 (0.89-0.98) | 2.28E-03 | 0.168 |
| rs7220550 | *STAT3* | 17 | 40473435 | A/G | 0.20 | 0.61 | 0 | 0.93 (0.89-0.98) | 2.31E-03 | 0.168 |
| rs8064496 | *STAT3* | 17 | 40474864 | A/G | 0.18 | 0.37 | 8 | 0.93 (0.89-0.98) | 3.69E-03 | 0.180 |
| rs8081431 | *STAT3* | 17 | 40474880 | G/C | 0.18 | 0.39 | 4 | 0.93 (0.89-0.98) | 3.75E-03 | 0.180 |
| rs6503693 | *STAT5A* | 17 | 40450891 | G/T | 0.18 | 0.46 | 0 | 0.93 (0.88-0.97) | 1.39E-03 | 0.168 |
| rs28664107 | *STAT5A* | 17 | 40450908 | G/A | 0.18 | 0.49 | 0 | 0.93 (0.89-0.97) | 1.77E-03 | 0.168 |
| rs1053005 | *STAT5A* | 17 | 40465910 | T/C | 0.20 | 0.62 | 0 | 0.93 (0.89-0.98) | 1.98E-03 | 0.168 |
| rs1053023 | *STAT5A* | 17 | 40465616 | T/C | 0.20 | 0.62 | 0 | 0.93 (0.89-0.98) | 1.99E-03 | 0.168 |
| rs3198502 | *STAT5A* | 17 | 40462994 | G/T | 0.20 | 0.64 | 0 | 0.93 (0.89-0.98) | 2.09E-03 | 0.168 |
| rs9907453 | *STAT5A* | 17 | 40456010 | G/T | 0.18 | 0.55 | 0 | 0.93 (0.89-0.97) | 2.12E-03 | 0.168 |
| rs909056 | *STAT5A* | 17 | 40464722 | G/C | 0.17 | 0.29 | 18 | 0.93 (0.87-0.98) | 2.29E-03 | 0.168 |
| rs56329056 | *STAT5A* | 17 | 40457088 | T/C | 0.18 | 0.38 | 6 | 0.93 (0.89-0.98) | 2.38E-03 | 0.168 |
| rs59316565 | *STAT5A* | 17 | 40457074 | T/C | 0.18 | 0.38 | 5 | 0.93 (0.89-0.98) | 2.44E-03 | 0.168 |
| rs2002052 | *STAT5A* | 17 | 40458957 | C/T | 0.18 | 0.41 | 1 | 0.93 (0.89-0.98) | 2.48E-03 | 0.168 |
| rs2293154 | *STAT5A* | 17 | 40461003 | C/T | 0.18 | 0.41 | 1 | 0.93 (0.89-0.98) | 2.75E-03 | 0.168 |
| rs60288073 | *STAT5A* | 17 | 40457246 | G/C | 0.18 | 0.40 | 3 | 0.93 (0.89-0.98) | 2.94E-03 | 0.168 |
| rs1135669 | *STAT5A* | 17 | 40459737 | C/T | 0.18 | 0.39 | 4 | 0.93 (0.89-0.98) | 3.07E-03 | 0.168 |
| rs9906989 | *STAT5A* | 17 | 40455846 | G/T | 0.18 | 0.46 | 0 | 0.93 (0.89-0.98) | 3.08E-03 | 0.168 |
| rs2272087 | *STAT5A* | 17 | 40459562 | A/G | 0.18 | 0.39 | 4 | 0.93 (0.89-0.98) | 3.09E-03 | 0.168 |
| rs2293155 | *STAT5A* | 17 | 40460989 | A/G | 0.18 | 0.41 | 2 | 0.93 (0.89-0.98) | 3.09E-03 | 0.168 |
| rs12601982 | *STAT5A* | 17 | 40461674 | A/G | 0.18 | 0.39 | 4 | 0.93 (0.89-0.98) | 3.37E-03 | 0.172 |
| rs2847297 | *PTPN2* | 18 | 12797694 | A/G | 0.33 | 0.46 | 0 | 0.94 (0.91-0.98) | 1.75E-03 | 0.168 |
| rs2847282 | *PTPN2* | 18 | 12819820 | T/G | 0.31 | 0.57 | 0 | 0.94 (0.90-0.98) | 2.77E-03 | 0.168 |
| Chr, chromosome; EAF, effect allele frequency; FDR, false discovery rate; OR, odds ratio; SNP, single nucleotide polymorphism.  ^a^Reference allele/effect allele.  ^b^Fixed effect models were used when no heterogeneity was found between studies (Q test *P* > 0.10 and *I*^2^ < 25.0%); otherwise, random effect models were used. | | | | | | | | | | |

| **Supplementary Table S3**. Summary of the functional prediction results of the 112 SNPs in the TCPTP pathways with FDR < 0.20 *in silico* | | | | | |
| --- | --- | --- | --- | --- | --- |
| SNP | Gene | Chr. | Position (hg19) | SNPinfo | RegulomeDB Score |
| rs4660342 | *PIK3R3* | 1 | 46595082 | -- | 6 |
| rs7538978 | *PIK3R3* | 1 | 46505054 | -- | 1f |
| rs785498 | *PIK3R3* | 1 | 46592414 | -- | 6 |
| rs809774 | *PIK3R3* | 1 | 46560244 | -- | 1f |
| rs785508 | *PIK3R3* | 1 | 46527159 | -- | 6 |
| rs785509 | *PIK3R3* | 1 | 46527300 | -- | 1f |
| rs785513 | *PIK3R3* | 1 | 46538320 | -- | 6 |
| rs785518 | *PIK3R3* | 1 | 46568422 | -- | 6 |
| rs785501 | *PIK3R3* | 1 | 46579611 | -- | 5 |
| rs1707303 | *PIK3R3* | 1 | 46598273 | TFBS+Splicing | 4 |
| rs814168 | *PIK3R3* | 1 | 46591623 | -- | 6 |
| rs785500 | *PIK3R3* | 1 | 46593044 | -- | 6 |
| rs785504 | *PIK3R3* | 1 | 46595696 | -- | 6 |
| rs785506 | *PIK3R3* | 1 | 46540652 | -- | 1f |
| rs785486 | *PIK3R3* | 1 | 46575148 | -- | 6 |
| rs785512 | *PIK3R3* | 1 | 46534543 | -- | 6 |
| rs1707337 | *PIK3R3* | 1 | 46508769 | miRNA | 6 |
| rs785490 | *PIK3R3* | 1 | 46577124 | -- | 6 |
| rs9429186 | *PIK3R3* | 1 | 46539687 | -- | -- |
| rs1768807 | *PIK3R3* | 1 | 46510570 | -- | 1f |
| rs1613296 | *PIK3R3* | 1 | 46546852 | -- | 6 |
| rs1768802 | *PIK3R3* | 1 | 46547868 | -- | -- |
| rs1588663 | *PIK3R3* | 1 | 46548825 | -- | 6 |
| rs1768800 | *PIK3R3* | 1 | 46549188 | -- | 6 |
| rs1768801 | *PIK3R3* | 1 | 46549137 | -- | 6 |
| rs785507 | *PIK3R3* | 1 | 46541558 | -- | 6 |
| rs1768818 | *PIK3R3* | 1 | 46514286 | TFBS | 6 |
| rs1768817 | *PIK3R3* | 1 | 46514534 | TFBS | 6 |
| rs1707339 | *PIK3R3* | 1 | 46513179 | -- | 6 |
| rs1085240 | *PIK3R3* | 1 | 46523978 | -- | -- |
| rs785470 | *PIK3R3* | 1 | 46519622 | TFBS | 1a |
| rs785465 | *PIK3R3* | 1 | 46522577 | -- | 6 |
| rs785468 | *PIK3R3* | 1 | 46521517 | Splicing | 6 |
| rs1707317 | *PIK3R3* | 1 | 46510642 | -- | 1f |
| rs1768815 | *PIK3R3* | 1 | 46528603 | -- | 1f |
| rs785493 | *PIK3R3* | 1 | 46584859 | -- | 6 |
| rs2297883 | *PIK3R3* | 1 | 46511487 | -- | -- |
| rs1707338 | *PIK3R3* | 1 | 46511981 | -- | 6 |
| rs1707322 | *PIK3R3* | 1 | 46505147 | -- | 1f |
| rs1612419 | *PIK3R3* | 1 | 46546945 | -- | 6 |
| rs785469 | *PIK3R3* | 1 | 46521091 | -- | 6 |
| rs785497 | *PIK3R3* | 1 | 46591903 | -- | 6 |
| rs785463 | *PIK3R3* | 1 | 46524234 | -- | 6 |
| rs785466 | *PIK3R3* | 1 | 46521792 | -- | -- |
| rs785483 | *PIK3R3* | 1 | 46559171 | -- | 6 |
| rs785510 | *PIK3R3* | 1 | 46528618 | -- | 1f |
| rs785467 | *PIK3R3* | 1 | 46521559 | -- | 6 |
| rs2458400 | *PIK3R3* | 1 | 46530798 | -- | 6 |
| rs796773 | *PIK3R3* | 1 | 46564475 | -- | 5 |
| rs785519 | *PIK3R3* | 1 | 46568562 | -- | -- |
| rs785484 | *PIK3R3* | 1 | 46574015 | -- | 6 |
| rs785517 | *PIK3R3* | 1 | 46567535 | -- | 6 |
| rs785516 | *PIK3R3* | 1 | 46564758 | -- | 5 |
| rs785496 | *PIK3R3* | 1 | 46588006 | -- | 6 |
| rs785462 | *PIK3R3* | 1 | 46525751 | -- | 6 |
| rs11707731 | *ATR* | 3 | 142287627 | -- | 4 |
| rs68065420 | *ATR* | 3 | 142289863 | -- | -- |
| rs13156223 | *PIK3R1* | 5 | 67525540 | -- | 4 |
| rs706714 | *PIK3R1* | 5 | 67522851 | TFBS | 5 |
| rs10515070 | *PIK3R1* | 5 | 67525575 | -- | 4 |
| rs706713 | *PIK3R1* | 5 | 67522722 | -- | 3a |
| rs173701 | *PIK3R1* | 5 | 67512124 | -- | 4 |
| rs845559 | *EGFR* | 7 | 55247960 | -- | 5 |
| rs2692456 | *EGFR* | 7 | 55272826 | -- | 6 |
| rs2740762 | *EGFR* | 7 | 55261342 | TFBS | 5 |
| rs940805 | *EGFR* | 7 | 55251807 | -- | -- |
| rs845553 | *EGFR* | 7 | 55245717 | -- | 4 |
| rs845554 | *EGFR* | 7 | 55246664 | -- | 2b |
| rs1140475 | *EGFR* | 7 | 55266417 | Splicing | 4 |
| rs17172432 | *EGFR* | 7 | 55141317 | -- | 4 |
| rs12532468 | *EGFR* | 7 | 55248231 | -- | 5 |
| rs2075101 | *EGFR* | 7 | 55250026 | -- | 5 |
| rs13243364 | *EGFR* | 7 | 55248488 | -- | 5 |
| rs1050171 | *EGFR* | 7 | 55249063 | -- | 4 |
| rs6944906 | *EGFR* | 7 | 55251953 | -- | 5 |
| rs845560 | *EGFR* | 7 | 55250794 | -- | 6 |
| rs2283053 | *MET* | 7 | 116427019 | -- | 4 |
| rs2299440 | *MET* | 7 | 116416327 | -- | 5 |
| rs2073560 | *MET* | 7 | 116423161 | -- | 6 |
| rs13239139 | *MET* | 7 | 116435123 | -- | 6 |
| rs3807997 | *MET* | 7 | 116420558 | -- | 5 |
| rs35314984 | *MET* | 7 | 116431948 | -- | -- |
| rs34280975 | *MET* | 7 | 116430265 | -- | 2c |
| rs9909736 | *STAT3* | 17 | 40470342 | -- | -- |
| rs3809758 | *STAT3* | 17 | 40471980 | -- | 4 |
| rs9891867 | *STAT3* | 17 | 40468489 | -- | 6 |
| rs8066464 | *STAT3* | 17 | 40471101 | -- | 5 |
| rs8074524 | *STAT3* | 17 | 40469598 | -- | 4 |
| rs3744483 | *STAT3* | 17 | 40466438 | miRNA | 4 |
| rs9909659 | *STAT3* | 17 | 40473835 | -- | 5 |
| rs7220550 | *STAT3* | 17 | 40473435 | -- | 5 |
| rs8064496 | *STAT3* | 17 | 40474864 | -- | 4 |
| rs8081431 | *STAT3* | 17 | 40474880 | -- | 2b |
| rs6503693 | *STAT5A* | 17 | 40450891 | -- | 5 |
| rs28664107 | *STAT5A* | 17 | 40450908 | -- | 5 |
| rs1053005 | *STAT5A* | 17 | 40465910 | miRNA | 4 |
| rs1053023 | *STAT5A* | 17 | 40465616 | miRNA | 3a |
| rs3198502 | *STAT5A* | 17 | 40462994 | miRNA | 4 |
| rs9907453 | *STAT5A* | 17 | 40456010 | -- | 2b |
| rs909056 | *STAT5A* | 17 | 40464722 | -- | 2b |
| rs56329056 | *STAT5A* | 17 | 40457088 | -- | 5 |
| rs59316565 | *STAT5A* | 17 | 40457074 | -- | 5 |
| rs2002052 | *STAT5A* | 17 | 40458957 | -- | 6 |
| rs2293154 | *STAT5A* | 17 | 40461003 | -- | 4 |
| rs60288073 | *STAT5A* | 17 | 40457246 | -- | 6 |
| rs1135669 | *STAT5A* | 17 | 40459737 | Splicing | 4 |
| rs9906989 | *STAT5A* | 17 | 40455846 | -- | 4 |
| rs2272087 | *STAT5A* | 17 | 40459562 | -- | 4 |
| rs2293155 | *STAT5A* | 17 | 40460989 | -- | 4 |
| rs12601982 | *STAT5A* | 17 | 40461674 | -- | 3a |
| rs2847297 | *PTPN2* | 18 | 12797694 | -- | -- |
| rs2847282 | *PTPN2* | 18 | 12819820 | -- | 5 |


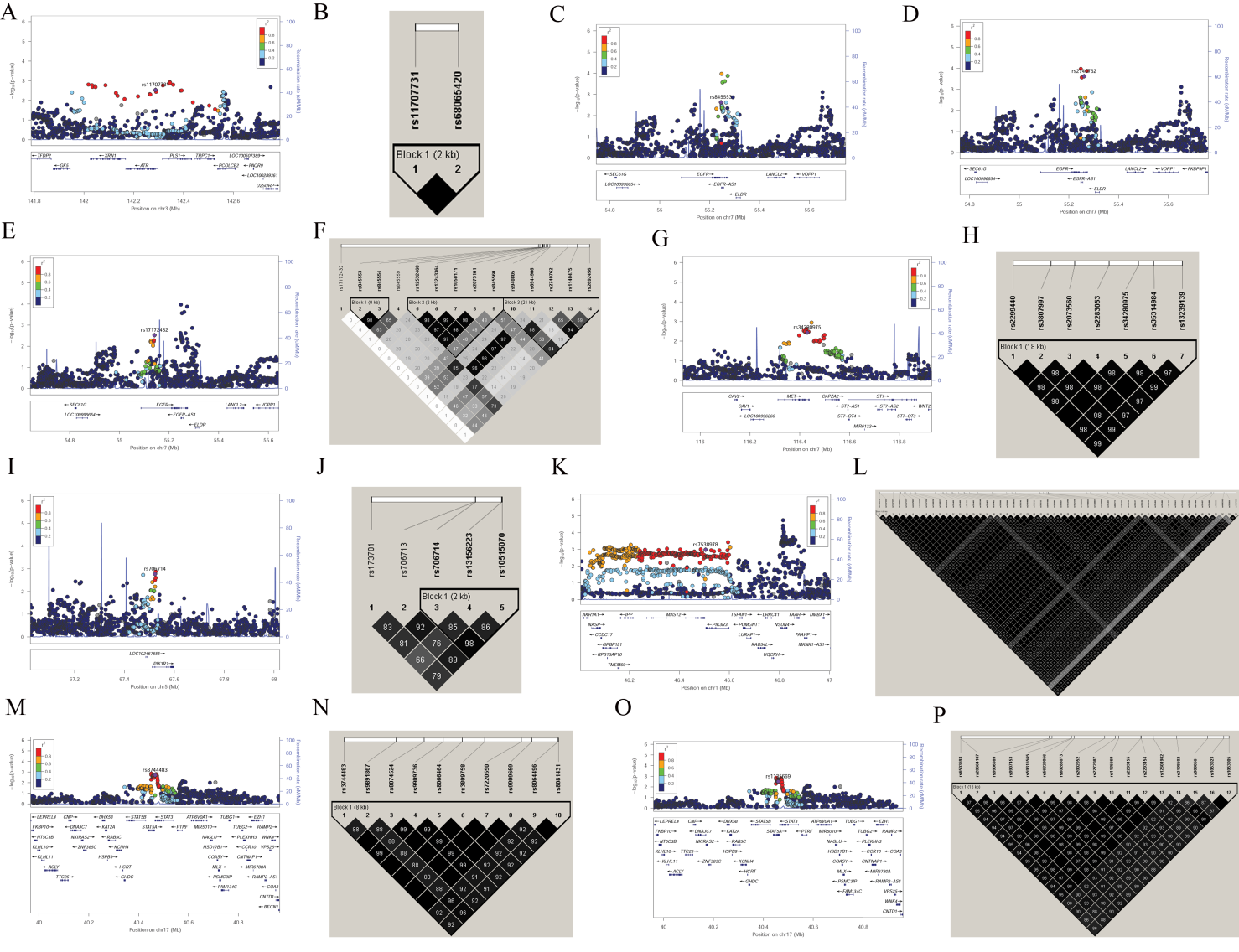


**Supplementary figure S1.** Regional association plots and linkage disequilibrium (LD) plots of the SNPs in 7 genes of TCPTP pathway. Data points are colored according to their level of LD with the SNP. Rs11707731 (A) in *ATR*; rs845553 (C), rs1140762 (D) and rs17172432 (E) in *EGFR*; rs34280975 (G) in *MET*; rs706714 (I) in *PIK3R1;* rs7538978 (K) in *PIK3R3;* rs3744483 (M) in *STAT3;* rs1135669 (O) in *STAT5A* with 500 kb up- and downstream of the gene region and LD plots of rs11707731 (B) in *ATR;* the SNPs (F) in *EGFR;* rs34280975 (H) in *MET*; rs706714 (J) in *PIK3R1;* rs7538978 (L) in *PIK3R3;* rs3744483 (N) in *STAT3;* rs1135669 (P) in *STAT5A* with FDR < 0.20. In A, C, D, E, G, I, K, M, O, the left-hand y-axis shows the association *P* value of each SNP, which is plotted as -log_10_ (*P*) against chromosomal base pair position; the right-hand y-axis shows the recombination rate estimated from the hg19/1000 Genomes European population.


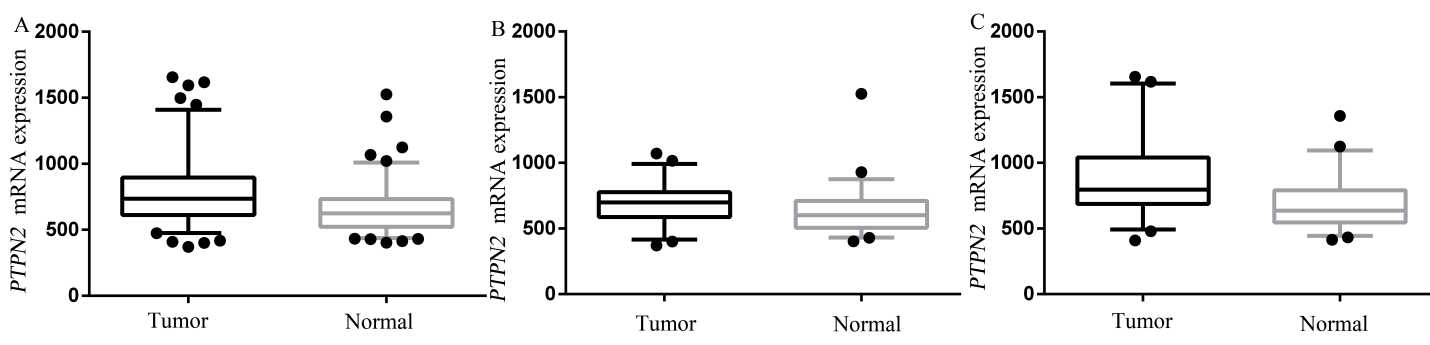


**Supplementary figure S2.** The mRNA expression of *PTPN2* in the 109 paired lung cancer and normal adjacent tissue samples from the TCGA database (A, overall, *P* = 1.515E-05; B, adenocarcinoma, *P* = 0.028; C, squamous cell carcinoma, *P* = 1.541E-04).

**Supplementary file**

**Study populations in TRICL-ILCCO**

*IARC GWAS.* The IARC GWAS comprised 3,062 lung cancer cases and 4,455 controls derived from five case-control studies: (i) the Carotene and Retinol Efficacy Trial (CARET) cohort; (ii) the Central Europe multicenter hospital-based case-control study; (iii) the hospital-based case-control study from France; (iv) the hospital-based case-control lung cancer study from Estonia; and (v) the population-based HUNT2/Tromsø IV lung cancer studies. Patient and control DNAs were derived from EDTA–venous blood samples. The patients with lung cancer were classified according to ICD-O-3: SQ: 8070/3, 8071/3, 8072/3, 8074/3; AD: 8140/3, 8250/3, 8260/3, 8310/3, 8480/3, 8560/3, 8251/3, 8490/3, 8570/3, 8574/3; with tumors with overlapping histologies being classified as mixed. After applying standardized quality-control procedures, 2,533 cases and 3,791 controls were included in the current analysis.

*NCI GWAS.* Details of the NCI GWAS have been reported previously. Briefly, the study comprised samples from four series: (i) the Environment and Genetics in Lung cancer Etiology (EAGLE) study, a population-based case-control study of 2,100 lung cancer cases and 2,120 healthy controls enrolled in Italy between 2002 and 2005, in which cancers were classified according to the ICD-O coding for histology and grading and histology of ~10% of tumors was confirmed by an independent pathologist from the NCI; (ii) the α-Tocopherol, β-Carotene Cancer Prevention Study (ATBC), a randomized primary prevention trial of 29,133 male smokers enrolled in Finland between 1985 and 1993, in which ICD-O-2 and ICD-O-3 were used to classify tumors and cases diagnosed between 1985 and 1999 had histology reviewed by at least one pathologist (after 1999, histological coding (ICD-O-2 and ICD-O-3) was derived from the Finnish Cancer Registry); (iii) the Prostate, Lung, Colon, Ovary Screening Trial (PLCO), a randomized trial of 150,000 individuals enrolled in 10 US study centers between 1992 and 2001, in which ICD-O-2 was used to classify tumors and quality assurance measures included reabstraction of 50 lung cancer diagnoses per year; and (iv) the Cancer Prevention Study II Nutrition Cohort (CPS-II), a cohort study of approximately 184,000 individuals enrolled by the American Cancer Society between 1992 and 1993 in 21 US states, of which 109,379 provided a blood (36%) or buccal (64%) sample between 1998 and 2003 and tumor histology was abstracted from Certified Tumor Registrars and coded using WHO ICD-O-2 and ICD-O-3. In this study, quality assurance was done by reabstracting 10% of all cancer diagnoses per year. After initial data quality control, the NCI GWAS included 5,739 cases and 5,848 controls; however, an additional 26 cases and 112 controls were excluded because of changes in case status and further quality-control filtering. The current meta-analysis included 5,713 lung cancer cases and 5,736 controls from the NCI GWAS.

*ICR GWAS.* The ICR GWAS comprised 1,952 cases (1,166 male; mean age at diagnosis 57 years, s.d. 6 years) with pathologically confirmed lung cancer ascertained through the Genetic Lung Cancer Predisposition Study (GELCAPS) conducted between March 1999 and July 2004. All cases were British residents and were self-reported to be of European ancestry. To ensure that data and samples were collected from *bona fide* lung cancer cases and avoid issues of bias from survivorship, only incident cases with histologically or cytologically (if not AD) confirmed primary disease were ascertained. Tumors from patients were classified according to ICD-O3: specifically, SQ: 8070/3, 8071/3, 8072/3, 8074/3; AD: 8140/3, 8250/3, 8260/3, 8310/3, 8480/3, 8560/3, 8251/3, 8490/3, 8570/3, 8574/3; with tumors with overlapping histologies being classified as mixed. Patient DNA was derived from EDTA–venous blood samples using conventional methodologies. Genotype frequencies were compared with publicly accessible data generated by the UK Wellcome Trust Case-Control Consortium 2 (WTCCC2) study of individuals from the 1958 British Birth Cohort (58BC), and blood service was typed using Illumina Human1.2M-Duo Custom_v1 Array BeadChips.

*MDACC GWAS.* Cases and controls were ascertained from a case-control study at the University of Texas MD Anderson Cancer Center conducted between 1997 and 2007. Cases were newly diagnosed patients with histologically confirmed lung cancer presenting at MD Anderson Cancer who had not previously received treatment other than surgery. Clinical and pathological data were abstracted from patient medical records, and lung cancer histology was coded according to the major histological groups. Specifically, as per ICD-O-2, these groups were SQ: 8070/3; AD: 8140/3, 8250/3, 8260/3, 8310/3, 8480/3, 8251/3, 8490/3. Only patients with predominantly or wholly AD or SQ cancers were included; those with mixed histology or unspecified lung cancers were excluded from the study. Controls were healthy individuals seen for routine care at Kelsey-Seybold clinics in the Houston metropolitan area. Controls were frequency matched to cases according to smoking behavior, age in 5-year categories, ethnicity and sex. Former smoking controls were further frequency matched to former smoking cases according to the number of years since smoking cessation (in 5-year categories). After applying quality controls, data were available on 1,150 cases and 1,134 controls.

*Heidelberg-EPIC (GLC).* This study comprised 1,253 Heidelberg-EPIC controls and 1,362 lung cancer cases from the Heidelberg lung cancer study recruited between 1994 and 1998 and between 1996 and 2007, respectively. Details of the Heidelberg-EPIC controls and the Heidelberg lung cancer study have been described previously. All subjects were aged 18 years or older, and information on lifestyle risk factors and medical and family history was collected through interviews based on standardized questionnaires. The EPIC Lung and the Heidelberg-EPIC studies were performed independently with no sample overlap with those analyzed as part of the IARC replication series. Histological classification of tumors was obtained from pathology reports, where it was recorded by a staff pulmonary pathologist according to WHO guidelines. Blood samples from patients with malignant lung disease categorized as follows were included: AD, SCLC, NSCLC, LCC, carcinoid, mixed lung tumors or mixed without SCLC. The above-described EPIC Lung and Heidelberg-EPIC studies were performed independently with no sample overlap. Genotypes for SNPs showed no significant departure from HWE, with the exception of rs13314271 in cases.

*Harvard.* For the Harvard Lung Cancer Susceptibility Study, details of participant recruitment have been described previously. Replication was based on data derived from 1,000 cases and 1,000 controls genotyped using Illumina HumanHap610-Quad arrays. Cases were patients aged >18 years with newly diagnosed, histologically confirmed primary NSCLC. Controls were healthy non–blood related family members and friends of patients with cancer or with cardiothoracic conditions undergoing surgery. The histological classification of lung tumors was performed by two staff pulmonary pathologists at Massachusetts General Hospital according to ICD-O-3: specifically, AD: 8140/3, 8250/3, 8260/3, 8310/3, 8480/3 8560/3; LCC: 8012/3, 8031/3; SQ: 8070/3, 8071/3, 8072/3, 8074/3; other NSCLC: 8010/3, 8020/3, 8021/3, 8032/3, 8230/3. Unqualified samples were excluded if they fit the following quality-control criteria: (i) overall genotype completion rates <95%; (ii) gender discrepancies; (iii) unexpected duplicates or probable relatives (based on a pairwise identity-by-state value of PI_HAT in PLINK >0.185); (iv) heterozygosity rates >6 times the s.d. from the mean; or (v) individuals evaluated to be of non-European ancestry (using HapMap release 23 including the JPT, CEPH, CEU and YRI populations as a reference). Unqualified SNPs were excluded when they fit the following quality-control criteria: (i) SNPs were not mapped on autosomes; (ii) SNPs had a call rate <95% in all GWAS samples; (iii) SNPs had MAF <0.01; or (iv) the genotype distributions of SNPs deviated from those expected by Hardy-Weinberg equilibrium (*P* < 1.0 × 10^−6^). After applying these prespecified quality controls, genotype data were available for 984 cases and 970 controls.

*deCODE.* The Icelandic lung cancer study has been described previously. The primary source of information on the Icelandic lung cancer cases is the Icelandic Cancer Registry (ICaR), which covers the entire population of Iceland (<http://www.cancerregistry.is/krabbameinsskra/indexen.jsp?id=summary>). The sources of data in the ICaR are all pathology and hematology laboratories and all hospital departments and health care facilities in the country. ICaR registration is based on the ICD system and includes information on histology (systemized nomenclature of medicine, SNOMED). ICaR registration also uses the ICD-O system, which takes histology diagnosis into account. Over 94% of diagnoses in the ICaR have histological confirmation. Briefly, according to the ICaR, a total of 4,252 patients were diagnosed with lung cancer from January 1, 1955 to December 31, 2010. Recruitment of both prevalent and incident cases was initiated in 1998, the recruitment is ongoing and DNA samples from lung cancer cases are subjected to whole-genome genotyping as they are collected. The controls used in this study consisted of individuals from other GWAS that were age and sex matched to cases, with no individual disease group accounting for >10% of all controls. Samples were assayed with the Illumina HumanHap300, HumanCNV370, HumanHap610, HumanHap1M, HumanHap660, Omni-1, Omni 2.5 or Omni Express bead chips at deCODE genetics. SNPs were excluded if they had (i) a yield <95%, (ii) MAF < 1% in the population, (iii) deviation from Hardy-Weinberg equilibrium (HWE; *P* < 10^−6^), (iv) inheritance error rate (>0.001) or (v) if there was a substantial difference in allele frequency between chip types (in which case the SNP was removed from a single chip type if that resolved the difference, but if it did not then the SNP was removed from all chip types). All samples with a call rate of <97% were removed from the analysis. The Icelandic sample set is drawn from the Icelandic population, which is a small homogeneous founder population with almost no detectable population substructure. Thus, there was no need to adjust for such substructure in the association analysis. In addition, the comprehensive Icelandic genealogy database allowed us to exclude individuals not of Icelandic origin from the analysis. SNP genotypes were phased using the method of long-range phasing[51](http://www.nature.com/ng/journal/v46/n7/full/ng.3002.html#ref51); for the HumanHap series of chips, 304,937 SNPs were used for long-range phasing, whereas for the Omni series of chips, 564,196 SNPs were used. An initial imputation step was carried out on each chip series separately to create a single harmonized, long-range phased genotype data set consisting of 707,525 SNPs for 95,085 Icelandic individuals. Two sets of genotypes were imputed into this data set with methods previously described: (i) genotypes for about 38 million variants using the 1000 Genomes phase I integrated variant set (v3) as training set and (ii) genotypes for about 34 million variants identified in 2,230 whole genome–sequenced Icelanders. The first set of imputed genotypes was used for replicating the association with variants in the 5p15.33, 9p21 and 12q13.33 regions using IMPUTE (v2.1.1) to perform the case-control analysis. The second set was used when testing the relationship between the p.Lys3326X and c.999del5 genotypes and risk of different cancer types in the Icelandic population using a method that allowed including individuals that had not been chip typed but for whom genotype probabilities were imputed using methods of familial imputation.

*Toronto.* This study was conducted in the greater Toronto area from 2008 to 2013. Lung cancer cases were recruited at the hospitals in the network of the University of Toronto. Controls were selected randomly from individuals registered in the family medicine clinics databases and were frequency matched with cases on age and sex. All subjects were interviewed, and information on lifestyle risk factors, occupational history and medical and family history was collected using a standard questionnaire. Tumors were centrally reviewed by the reference pathologist (a member of the International Association for the Study of Lung Cancer (IASLC) committee) and a second pathologist in the University Health Network. If the reviews conflicted, a consensus was arrived at after discussion. Coding of histology was based on 2001 WHO/IASLC. After applying standardized quality control procedures and restricting the data to participants with self-reported European ancestry, data and samples were available on 1,084 cases and 966 controls. The genotype distributions of genotypes for each of the SNPs typed in replication showed no significant departure from HWE.
